# Inhibition of Plasma Kallikrein by a Highly Specific Active Site Blocking Antibody

**DOI:** 10.1074/jbc.M114.569061

**Published:** 2014-06-26

**Authors:** Jon A. Kenniston, Ryan R. Faucette, Diana Martik, Stephen R. Comeau, Allison P. Lindberg, Kris J. Kopacz, Gregory P. Conley, Jie Chen, Malini Viswanathan, Niksa Kastrapeli, Janja Cosic, Shauna Mason, Mike DiLeo, Jan Abendroth, Petr Kuzmic, Robert C. Ladner, Thomas E. Edwards, Christopher TenHoor, Burt A. Adelman, Andrew E. Nixon, Daniel J. Sexton

**Affiliations:** From the ‡ Dyax Corp., Burlington, Massachusetts 01803,; § Beryllium, Bainbridge Island, Washington 98110, and; ¶ Biokin, Watertown, Massachusetts 02472

**Keywords:** Antibody Engineering, Enzyme Inhibitor, Inflammation, Kallikrein, Protease

## Abstract

Plasma kallikrein (pKal) proteolytically cleaves high molecular weight kininogen to generate the potent vasodilator and the pro-inflammatory peptide, bradykinin. pKal activity is tightly regulated in healthy individuals by the serpin C1-inhibitor, but individuals with hereditary angioedema (HAE) are deficient in C1-inhibitor and consequently exhibit excessive bradykinin generation that in turn causes debilitating and potentially fatal swelling attacks. To develop a potential therapeutic agent for HAE and other pKal-mediated disorders, we used phage display to discover a fully human IgG1 monoclonal antibody (DX-2930) against pKal. *In vitro* experiments demonstrated that DX-2930 potently inhibits active pKal (*K_i_* = 0.120 ± 0.005 nm) but does not target either the zymogen (prekallikrein) or any other serine protease tested. These findings are supported by a 2.1-Å resolution crystal structure of pKal complexed to a DX-2930 Fab construct, which establishes that the pKal active site is fully occluded by the antibody. DX-2930 injected subcutaneously into cynomolgus monkeys exhibited a long half-life (*t*_½_ ∼12.5 days) and blocked high molecular weight kininogen proteolysis in activated plasma in a dose- and time-dependent manner. Furthermore, subcutaneous DX-2930 reduced carrageenan-induced paw edema in rats. A potent and long acting inhibitor of pKal activity could be an effective treatment option for pKal-mediated diseases, such as HAE.

## Teaser

**Background:** Unregulated plasma kallikrein proteolytic activity can result from C1-inhibitor deficiency, causing excessive and potentially fatal edema.

**Results:** The antibody DX-2930 potently and specifically inhibits plasma kallikrein and exhibits a long plasma half-life.

**Conclusion:** An antibody protease inhibitor can lead to potent and specific bioactivity.

**Significance:** DX-2930 could be an effective therapeutic for the prophylactic inhibition of plasma kallikrein-mediated diseases.

## Introduction

**FIGURE 1.**
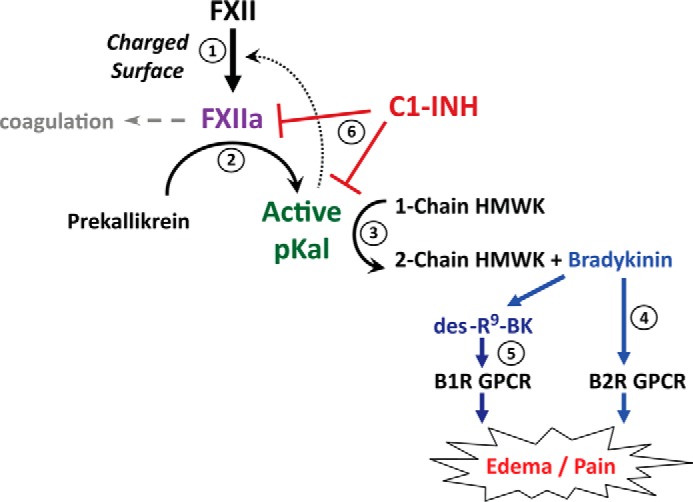
**Activation cycle of the kallikrein-kinin enzymatic pathway.** Autoactivation of the proenzyme form of factor XII to XIIa (*step 1*) initiates a proteolytic cascade that activates pKal (*step 2*), which in turn cleaves HWMK to release bradykinin (*step 3*) (in addition to the positive feedback activation of more FXII). Bradykinin subsequently binds and activates the bradykinin B_2_ receptor (*step 4*), thereby initiating an intracellular signaling cascade that ultimately results in swelling and pain in the affected tissues. Exoprotease activity on bradykinin generates des-Arg^9^bradykinin, which signals through the bradykinin B_1_ receptor (*step 5*). Individuals with HAE are deficient in C1-INH (*step 6*), the major inhibitor of active pKal and FXIIa, and subsequently exhibit unpredictable attacks of bradykinin-mediated pain and edema.

The plasma contact system is driven by the coordinated action of coagulation factor XII (FXII),^2^ pKal, and HMWK to generate proteolytically activated mediators of vascular biology, thrombosis, fibrinolysis, angiogenesis, and inflammation (1). Positioned at the interface between coagulation and inflammation, the contact system initiates intrinsic pathway coagulation and activates the plasma kallikrein-kinin system (KKS) by conversion of prekallikrein to active pKal. pKal is a serine protease that cleaves HMWK at two sites to liberate bradykinin, a nonapeptide agonist of the constitutive bradykinin B2 G-protein-coupled receptor and via its des-Arg^9^ bradykinin metabolite, an agonist of the inducible bradykinin B1 G-protein-coupled receptor (Fig. 1) (2). Activation of these receptors leads to the production of mediators of inflammation, vasodilation, angiogenesis, and pain (2).

Pathophysiologic *in vivo* triggers of contact system activation in human disease have been difficult to identify. However, the system has been shown to be activated *in vitro* and in preclinical disease models by numerous agents that include mast cell heparin (3), misfolded or aggregated proteins (4), sub-endothelial matrix proteins such as collagens and laminin (5–7), extracellular RNA (8), polyphosphate from platelets or bacteria (9, 10), fibrin (11), bacterial infection (12), ventricular assist devices (13), and following exposure of plasma to polyanionic substances such as kaolin, dextran sulfate, phospholipids, or surfaces such as glass (1). Furthermore, there are reports that the contact system is activated in plasma obtained from patients with diseases such as rheumatoid, psoriatic, or osteoarthritis (14), ulcerative colitis (15), systemic lupus erythematosus (16), psoriasis (16), diabetic retinopathy, and macular edema (17, 18), sepsis (19), systemic amyloidosis (4), sickle cell disease (20), Alzheimer disease (21), anaphylaxis (3), brain ischemia and edema (22), cirrhosis (23), and hereditary angioedema with C1-inhibitor deficiency (HAE-C1INH) (24–26).

HAE-C1INH is a rare disease caused by unregulated activation of the KKS that clinically manifests as intermittent attacks of subcutaneous or mucosal edema affecting the face, larynx, gastrointestinal tract, limbs, or genitalia (27). Affected individuals harbor autosomal dominant mutations in the C1-INH gene (SERPING1) that lead to a deficiency in either total (type I HAE-C1INH) or functional (type II HAE-C1INH) C1-INH protein, a protease inhibitor of the serpin superfamily that regulates the contact, coagulation, fibrinolytic, and complement pathways (28, 29). During an HAE attack, reduced levels of functional C1-INH are insufficient to inhibit its key targets, both FXIIa and pKal (Fig. 1), thereby causing the overproduction of bradykinin and ultimately the pain and swelling characteristic of this disease.

Clinically, the role of the plasma KKS in HAE-C1INH pathophysiology has been demonstrated by the approval of therapeutics that inhibit this pathway. For treatment of acute attacks, these include C1-INH replacement, selective inhibition of pKal proteolytic activity with the Kunitz domain inhibitor ecallantide, or bradykinin B2 receptor antagonism with the bradykinin peptidomimetic icatibant (30). Prophylactic treatments include chronic C1-INH replacement, which is limited by the need for frequent intravenous administration that can lead to the placement of an indwelling catheter. C1-INH therapy is also associated with a risk for serious thromboembolic events (31). Oral androgens are an alternative prophylactic option that are more convenient to administer but are associated with serious toxicities and morbidities from unwanted androgenizing effects, particularly in women, as well as hepatic adenomas with malignant potential (32, 33). Furthermore, despite chronic treatment with existing prophylactic agents, breakthrough attacks still occur (34). Thus, there remains an unmet medical need for a long lasting, convenient, safe, and effective prophylactic therapeutic to treat HAE-C1INH.

Given the potential for target specificity and long serum half-life with antibody therapeutics, we used phage display to select a potent and highly specific human antibody inhibitor of pKal, DX-2930. Here, we describe the discovery and biochemical characterization of DX-2930, including the x-ray structure of the Fab·pKal complex, which provides a rationale for the specificity and effectiveness of DX-2930. Moreover, preclinical studies reveal DX-2930 to exhibit a prolonged half-life in circulation, and we further demonstrate that this translates into a prolonged capacity to inhibit the KKS. Taken together, data presented here support the potential of DX-2930 for the prophylactic inhibition of pKal-mediated edema and provide a highly specific reagent tool to investigate the effects of targeted pKal inhibition in complex biological samples containing highly homologous proteases.

## EXPERIMENTAL PROCEDURES

### 

#### 

##### Proteins

Full-length human plasma kallikrein (prekallikrein and activated pKal forms), 1-chain kininogen (intact kininogen), and 2-chain kininogen (treated with pKal to excise bradykinin) were obtained from Enzyme Research Laboratories. C1-INH and pKal orthologues from rat, cynomolgus monkey, and rabbit plasma were purified by Athens Research and Technology. The pKal catalytic domain variant used in crystallographic studies contained three deglycosylation (N396E, N453E, and N494E) and two cysteine (C383S and C503S) mutations and were purified as described previously (35).

##### Specificity

DX-2930 specificity screen measurements used the following 20 human serine proteases. Recombinant complement component C1s (R & D Systems) was assayed using 10 μm dabcyl-Ser-Leu-Gly-Arg-Lys-Ile-Gln-Ile-EDANS (where dabcyl is 4-((4-(dimethylamino)phenyl)azo)benzoyl and EDANS is 5-[(2-aminoethyl)amino]naphthalene-1-sulfonic acid) as substrate in Buffer A (25 mm Tris, pH 8.0, 100 mm NaCl, 0.01% Brij35). Factor XIIa (Enzyme Research Labs) was assayed using 10 μm Pro-Phe-Arg-AMC (where AMC is 7-amino-4-methylcoumarin) as substrate in Buffer B (20 mm Tris-HCl, pH 7.5, 150 mm NaCl, 1 mm EDTA, 0.1% PEG-8000, and 0.1% Triton X-100). Activated protein C (Haematologic Technologies, Inc.) was assayed using 50 μm Boc-d-VLR-ANSNH-C4H9 (where ANSNH is 6-amino-1-naphthalene-sulfonamide) as substrate in Buffer C (100 mm Tris-HCl, pH 8.0, 50 mm NaCl, 10 mm CaCl_2_, 0.025% CHAPS). Cathepsin G (Biomol) was assayed using 10 μm succinyl-Ala-Ala-Pro-Phe-AMC as substrate in Buffer C. Factor VIIa (Biomol) was assayed using 10 μm Z-Val-Val-Arg-AMC as substrate in Buffer A. Factor Xa (Biomol) was assayed using 10 μm CH_3_SO_2_-d-CHA-Gly-Arg-AMC-AcOH as substrate in Buffer A containing 0.25 mg/ml bovine serum albumin (BSA). Factor XIa (Haematologic Technology Inc.) was assayed using 10 μm *t*-butyloxycarbonyl-Glu(benzyl ester)-Ala-Arg-MCA (where MCA is 4-methylcoumaryl-7-amide) as substrate in Buffer A. Recombinant granzyme B (Biomol) was assayed using 10 μm Ac-Ile-Glu-Pro-Asp-AMC as substrate in Buffer A. Recombinant hepsin (R & D Systems) was assayed using 10 μm Pro-Phe-Arg-AMC as substrate in Buffer B. Recombinant matriptase (R & D Systems) was assayed using 10 μm Pro-Phe-Arg-AMC as substrate in Buffer B. Neutrophil elastase (Athens Research and Technology) was assayed using 100 μm *N*-methoxysuccinyl-Ala-Ala-Pro-Val-AMC as substrate in Buffer B. Plasmin (Haematologic Technology Inc.) was assayed using 10 μm H-d-CHA-Ala-Arg-AMC as substrate in Buffer A. Thrombin α (Enzyme Research Labs) was assayed using 10 μm H-d-CHA-Ala-Arg-AMC as substrate in Buffer A containing 2.5 mm CaCl_2_ and 1 mg/ml BSA. Recombinant tissue kallikrein 1 (R & D Systems) was assayed using 10 μm Pro-Phe-Arg-AMC as substrate in Buffer B. Recombinant tissue kallikrein 2 (36) was assayed using 10 μm Pro-Phe-Arg-AMC as substrate in Buffer B. Recombinant tissue kallikrein 5 (R & D Systems) was assayed using 10 μm Z-Val-Val-Arg-AMC as substrate in Buffer A. Recombinant tissue kallikrein 12 (R & D Systems) was assayed using 10 μm Val-Pro-Arg-AMC as substrate in Buffer C. Trypsin (Athens Research and Technology) was assayed using 10 μm Pro-Phe-Arg-AMC as substrate in Buffer B. Recombinant tissue plasminogen activator (Sigma) was assayed using 10 μm Z-Gly-Pro-Arg-AMC as substrate in Buffer A containing 1% BSA. Urokinase (Sigma) was assayed using 10 μm benzyl-Ala-Gly-Arg-AMC as substrate in Buffer A.

##### Phage Display Selection and Primary Screening

Human antibodies against pKal were obtained by panning an antibody phage display library (37) against biotinylated pKal immobilized on streptavidin-coated magnetic beads (Dynal, M280). Selections were performed as described (38), including positive selections with decreasing amounts of biotinylated human or rat pKal and negative selections with untreated streptavidin beads and biotinylated prekallikrein. Phage isolates were screened by ELISA (streptavidin-immobilized pKal with detection by anti-M13 coat protein VIII), positive hits were DNA-sequenced, and unique Fabs were batch-processed for *Escherichia coli* expression as isolated Fab fragments from the pMID21 vector as described (38). An affinity maturation library was constructed around the lead Fab (given its inhibition of both human and rat pKal) by employing mixed nucleotide synthesis of heavy chain variable complementary determining region 3 (HV-CDR3) where the nominal base was present at 85% and each of the others at 5%.

##### Antibody Expression and Purification

High throughput production of *E. coli*-secreted Fabs and their subsequent purification by protein A-Sepharose followed previously reported protocols (38). The DX-2930 Fab construct generated for crystallization studies was produced as follows. The DNA sequence for the DX-2930 Fab region was codon-optimized and cloned into the pJexpress 401 expression vector by DNA2.0. SS320 TG1 *E. coli* cells (Lucigen) were transformed with the Fab expression vector and grown in 10 ml of TBDry media containing 2% glucose and 25 μg/ml kanamycin for 18 h at 30 °C. 10 liters of TBDry media containing 0.1% glucose and 25 μg/ml kanamycin was then inoculated with 10 ml of the starter culture (1:1000 ratio) and grown for 20 h at 30 °C. Cells were harvested by centrifugation (15 min at 2000 × *g*); the pellet resuspended in 10 liters with TBDry media containing 25 μg/ml kanamycin, 1 mm isopropyl 1-thio-β-d-galactopyranoside, and 0.5% Triton X-100, and incubated an additional 20 h at 30 °C with shaking. The culture was finally harvested by spinning at 9000 × *g* for 30 min and filtered through a Corning 0.22-μm PES filter. The resulting culture supernatant containing secreted Fab protein was subjected to a three-step chromatography purification. Fab product was first captured by immunoaffinity protein A resin (RepliGen). 80 ml of immobilized protein A resin was added to the 10 liters of filtered supernatant and incubated with rocking for 18 h at 4 °C. Resin was captured in a fritted column and extensively washed to remove host cell-related impurities and reduce endotoxin level. Washes successively included 10 column volumes (cv) of PBS, 20 cv of Fab Wash Buffer (50 mm sodium acetate, 0.5 m sodium chloride, pH 5.0), 50 cv of PBS with 0.1% Triton X-100, and then a final wash with 50 cv PBS. Soluble Fab (sFab) was then eluted from protein A with 6 cv of Fab elution buffer (50 mm sodium phosphate, 150 mm sodium chloride, pH 2.5) and instantly neutralized to pH 6.0 with 2 cv equivalents of 1 m HEPES, pH 7.2. Neutralized protein A eluate was loaded onto PBS pre-equilibrated mercaptoethylpyridine HyperCel resin (Pall Lifesciences) and incubated overnight at 4 °C with rocking. Mercaptoethylpyridine resin was then washed with 10 cv of PBS, followed by step elutions with 50 mm sodium acetate solutions at varying pH values from pH 5 to pH 3. The major elution peak at pH 5 was collected and directly loaded onto a POROS HS 50 strong CEX resin (Invitrogen) pre-equilibrated with 50 mm sodium acetate, pH 4.5 (“CEX Buffer”), washed first with the same CEX Buffer, and then an additional wash with CEX Buffer + 250 mm NaCl. Fab was subsequently eluted with CEX Buffer + 350 mm NaCl. Purified Fab product was exchanged into a buffer containing 50 mm citrate/phosphate, 100 mm NaCl, pH 6.0, through an ultrafiltration/diafiltration process using a Millipore Amicon Ultra concentration unit (10,000-kDa molecular mass cutoff). Recombinant Fab fragments were reformatted into full-length human IgG1 antibodies (f-allotype) (39), expressed as stable transfections in CHO cells using a fed-batch fermentation strategy, and purified as previously described (40) into formulation buffer (100 mm citrate/phosphate buffer, pH 6.0, 50 mm NaCl, 2% (w/v) trehalose, and 0.01% Tween 80).

##### Enzyme Inhibition Analysis

**FIGURE 2.**
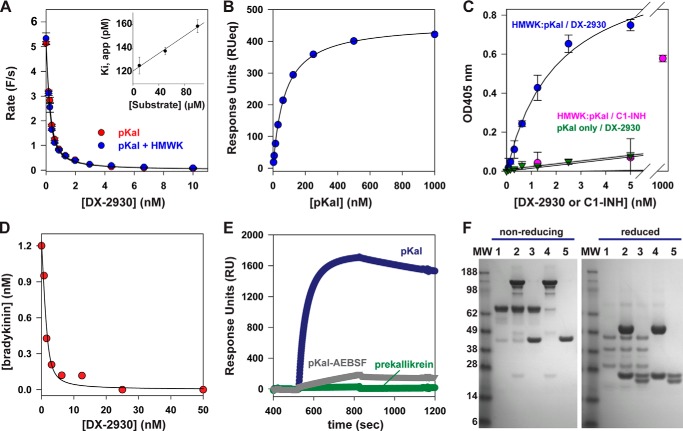
**DX-2930 potently inhibits pKal proteolytic activity.** *A,* increasing concentrations of DX-2930 were incubated with full-length pKal until equilibrium was achieved, and the rate of pKal-mediated proteolysis of the peptide PFR-AMC was measured by fluorescence changes over time (*F/s*). Inhibition data were fit to Equation 1. The *inset* demonstrates the linear increase in *K*_*i*_^app^ for DX-2930 interaction with pKal alone as a function of increasing substrate concentration. This behavior is consistent with a competitive inhibition mechanism (41), yielding an average *K_i_* = 0.120 ± 0.005 nm from Equation 2 (see under “Experimental Procedures”). A similar *K_i_* value was generated for pKal that was preincubated with 600 nm HMWK (*K_i_* = 0.115 ± 0.003 nm), an excess concentration of HMWK that promotes near-complete complex formation with pKal as shown in *B,* a plot of equilibrium biosensor response units (*RU*) with increasing concentrations of pKal flowed over a biosensor surface bearing immobilized HMWK. *C,* ELISA detection of DX-2930 binding to pKal complexed with HWMK that is HUVEC cell membrane-bound. Unlike C1-INH, DX-2930 can effectively target membrane-anchored pKal. *D,* DX-2930 also potently inhibits cleavage of the native pKal substrate HMWK and consequently prevents the release of detectable bradykinin, as assessed by a semi-quantitative ELISA that detects the bradykinin nonapeptide in solution. *Error bars* shown above are the standard deviation between replicates. *E,* characterization of DX-2930 binding specificity by surface plasmon resonance. The binding of 500 nm pKal, prekallikrein, and pKal-AEBSF to a DX-2930-coated biosensor surface was monitored over time. The loss of binding to both the prekallikrein and pKal-AEBSF samples suggests DX-2930 interacts with the pKal active site. *F,* Coomassie-stained SDS-PAGE analysis of DX-2930 IgG or Fab construct (both at 2 μm) incubated with 2 μm pKal for 30 min at 30 °C. No proteolysis of DX-2930 IgG or Fab is observed in either reducing (5 mm DTT) or nonreducing PAGE (no new fragments generated), confirming that the intimate binding of the DX-2930 HV-CDR3 loop with the pKal active site does not result in cleavage of the antibody. *Lane 1*, pKal only; *lane 2,* pKal and DX-2930; *lane 3,* pKal and DX-2930 Fab; *lane 4,* DX-2930 only; *lane 5,* DX-2930 Fab only.

Steady state inhibition measurements were performed as described previously (40). Briefly, pKal (nominal concentration of 1 nm as total protein) was incubated with different concentrations of inhibitor for 1 h at 30 °C in Buffer B (20 mm Tris-HCl, pH 7.5, 150 mm NaCl, 1 mm EDTA, 0.1% PEG-8000, and 0.1% Triton X-100) in a 96-well plate. The maximum final inhibitor concentration was 10 nm, followed by a 1.5-fold serial dilution (total 11 nonzero inhibitor concentrations) plus a control ([I]_0_ = 0). The reactions were initiated by the addition of H-Pro-Phe-Arg-AMC substrate (Sigma, catalogue no. P9273) at a final concentration of 10, 50, or 100 μm and monitored in a fluorescence plate reader (SpectraMax, Molecular Devices) with excitation and emission wavelengths at 360 and 480 nm, respectively. Initial reaction rates were determined by linear fit of raw experimental data traces (fluorescence *versus* time) as the slopes of the regression lines. The initial reaction rates, *v*, were fit to Equation 1.

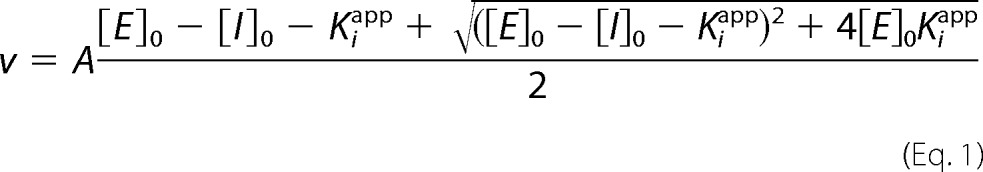
 In Equation 1, [*I*]_0_ is the inhibitor concentration; [*E*]_0_ is the active enzyme concentration; *K*_*i*_^app^ is the apparent inhibition constant; and *A* is an empirical constant equal to ϵ*_p_k*_cat_[S]_0_/([S]_0_ + *K_m_*), where ϵ*_p_* is the molar response coefficient of the reaction product; *k*_cat_ is the turnover number; [S]_0_ is the initial substrate concentration; and *K_m_* is the Michaelis constant. The adjustable parameters were *A*, *K*_*i*_^app^, and [*E*]_0._ Note that Equation 1 is Cha's variant (see Equation 14 in Ref. 41) of the rate equation initially derived by Morrison (42). In contrast with Morrison's original formulation, the enzyme concentration [*E*]_0_ does not appear in the denominator and instead is subsumed in the empirical constant *A*. This formulation significantly increases numerical stability in nonlinear regression for the following reason. In our particular case ([*E*]_0_ = 1 nm, *K*_*i*_^app^ ≈0.1 nm) the enzyme concentration [*E*]_0_ must be treated as an adjustable parameter, according to the theoretical principles explained elsewhere (43). However, during the iterative regression analysis, [*E*]_0_ could transiently attain values very near zero, which could cause numerical underflow (“division by zero”) in the original form of the Morrison equation (42). The Michaelis constant *K_m_* for the synthetic substrate was determined to be 315 ± 16 μm (data not shown). At a substrate concentration [S]_0_ = 10 μm, the factor 1 + [S]_0_/*K_m_* in Equation 2 (assuming competitive inhibition, see *inset* in Fig. 2*A*) is equal to 1.03.

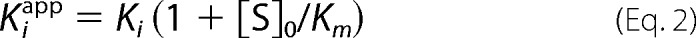
 Thus, in our particular case the apparent inhibition constants *K*_*i*_^app^ obtained in the regression analysis are identical to the true inhibition constant within 3%. The activity of 20 different human serine proteases listed above was observed in the absence or presence of 1 μm DX-2930 using synthetic chromogenic or fluorogenic substrates.

##### ELISA Measurement of pKal-mediated Bradykinin Formation

Following the incubation of human pKal (0.15 nm) with varying concentrations of DX-2930 for 1 h at 30 °C in Buffer B, 1-chain HMWK was added at a final concentration of 20 nm. After the reaction proceeded for 10 min at 30 °C, samples were applied to a 96-well 10-kDa molecular mass cutoff filter plate (Millipore MultiScreen with Ultracel-10 membrane), centrifuged at 400 × *g* in a table top centrifuge for 45 min to remove unreacted HMWK, and analyzed by an anti-bradykinin ELISA (Peninsula Laboratories) per the manufacturer's instructions.

##### ELISA Detection of pKal Binding to HUVEC

Human umbilical vein endothelial cells (Lonza) were seeded onto 96-well plates at ∼2 × 10^4^ cells/well, and incubated for 48 h. The wells were blocked with 3% BSA in Buffer D (20 mm HEPES, 150 mm NaCl, 2 mm CaCl_2_, 1 mm MgCl_2_, pH 7.5), washed, and then treated with Buffer E (Buffer D + 50 μm ZnSO_4_ and 0.5 mg/ml BSA) or HMWK (40 nm in Buffer E) for 1 h at 25 °C, followed by a 1-h incubation with 3 nm pKal or pKal pretreated with 1 mm 4-(2-aminoethyl) benzenesulfonyl fluoride hydrochloride (AEBSF, Thermoscientific). Varying concentrations of biotinylated DX-2930 were then added to the treated cells for 2 h, and bound antibody was detected by streptavidin-alkaline phosphatase and *p*-nitrophenyl phosphate additions.

##### Surface Plasmon Resonance

Surface plasmon resonance (SPR) measurements were performed using a BIAcore 3000 (GE Healthcare) with the detection temperature at 25 °C and HBS-P running buffer (10 mm HEPES, pH 7.4, 150 mm NaCl, and 0.005% surfactant P20). To measure kallikrein interactions with DX-2930, goat anti-human Fc fragment-specific IgG (Jackson ImmunoResearch) was immobilized by amine coupling on a CM5 sensor chip at ∼5000 response units (RU), and DX-2930 was subsequently captured by injecting a 5 nm antibody solution for 4 min at 5 μl/min. Reference surfaces were activated and blocked in mock amine coupling reactions. 500 nm prekallikrein, pKal, or pKal-AEBSF were injected for 5 min at 20 μl/min followed by a 5-min dissociation phase. Surfaces were regenerated with a 30-s pulse of 10 mm glycine, pH 1.5, at 100 μl/min. To measure the affinity of pKal to HMWK, 2-chain HMWK was immobilized by amine coupling on a CM5 chip at ∼2000 RU, and varying concentrations of pKal were flowed over the surface for 5 min at 20 μl/min, and dissociation followed for 5 min. Equilibrium RU values for each pKal concentration, [*E*], were fit to Equation 3 to obtain the observed dissociation equilibrium constant *K_D_*. Reference surface and chip regeneration followed the above procedures.

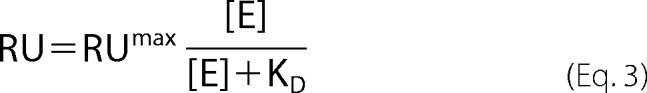


##### X-ray Crystallographic Structure of the DX-2930·pKal Complex

Purified DX-2930 Fab and the deglycosylated pKal catalytic domain variant (35) were incubated at 1:1 stoichiometry. The resulting complex (150 μm) in addition to Fab alone was diluted 1:1 in reservoir precipitant (Table 2) and crystallized by the hanging drop vapor diffusion method. Diffraction data from resulting crystals were collected with a Rigaku FR-E+ SuperBright x-ray generator with a Saturn 944+ CCD detector and VariMax HF optics (complex) and at the Argonne National Laboratory (APS) beam line (Fab alone, refer to Table 2). The complex structure was solved by molecular replacement in Phaser using separate constant and variable domains of a Fab model (derived from PDB 3IDX) and the pKal catalytic domain structure PDB 2ANY (35). The crystallographic model of the 2930 Fab determined from the pKal·2930 Fab complex structure was then used to help solve the Fab-only structure. The final models were obtained after iterative building/refinement in REFMAC/COOT (44–46).

##### Pharmacokinetic Properties of DX-2930 in Cynomolgus Monkey

Six male cynomolgus monkeys were assigned to two dose groups, each consisting of three animals. All animals (treated at WIL Research) were dosed on day 0, with group 1 receiving a single i.v. injection of 20 mg/kg DX-2930 (2 ml/kg of DX-2930 at 10 mg/ml) and Group 2 receiving a single s.c. injection of 20 mg/kg DX-2930 (also 2 ml/kg of DX-2930 at 10 mg/ml). Blood was collected from all animals in citrated plastic tubes at the following time points: predose, 5 min; 1, 4, 24, 48, 96, 168, 240, 336, 432, 504, 672, 840, and 1008 h post-dose. Plasma samples were analyzed using a qualified sandwich ELISA method. Briefly, 96-well microplates (Costar 3059) were coated with 1 μg/ml goat anti-human IgG (Bethyl Labs: pre-adsorbed with monkey IgG; diluted in 100 mm sodium carbonate, pH 9.4) by incubation at 4 °C overnight and subsequently blocked with 3% BSA in 1× PBS (incubated 2 h at 37 °C). Test sample and antibody standards were then diluted 1:10 with PBST-B (1× PBS, 0.1% Tween 20, and 3% BSA) and captured on the microplate by incubation for 2 h at 37 °C. Finally, bound IgG/DX-2930 was detected by the addition of 50 ng/ml HRP-labeled goat anti-human IgG (Bethyl Labs) diluted in PBST-B. Antibody standard dilutions were included to calculate the concentration of DX-2930 in sample plasma. From these data, the pharmacokinetic parameters were calculated using WinNonlin Professional Version 5.3 (Pharsight Inc.). All data were analyzed noncompartmentally.

##### Ex Vivo Analysis of HMWK Cleavage by pKal

Cynomolgus monkeys were subcutaneously injected with 25 and 50 mg/kg DX-2930, and blood was collected as above at WIL Research. Aliquots of the resulting citrated plasma samples were activated with 150 μg/ml kaolin for 10 min at 37 °C, diluted 1:20 in 1× PBS, and aliquots analyzed by Western blots blocked in PBST + 2% BSA and probed with 1:2000 HRP-labeled sheep anti-human kininogen polyclonal antibody (Cedarlane) diluted in PBST + 2% BSA.

##### Carrageenan-induced Paw Edema

Male Sprague-Dawley rats (180–200 g each, Harlan Laboratories) were divided into different treatment groups (*n* = 10 each group) and treated at Washington Biotechnology, Inc., with either an i.p. injection of Formulation Buffer, indomethacin-positive control (Sigma, 5 mg/kg prepared in 0.1 m NaHCO_3_ at a concentration of 2.5 mg/ml), or an s.c. injection of different doses of DX-2930. The i.p. and s.c. injections were administered 30 min and 24 h, respectively, prior to the injection of 0.1 ml of 1% λ carrageenan (FLUKA, prepared in distilled water) into the right hind paw. Paw volumes were measured by plethysmography before and 1, 2, 4, 6, and 8 h after carrageenan injection.

## RESULTS

### 

#### 

##### Discovery of DX-2930 by Antibody Phage Display and Affinity Maturation

Phage isolates were screened for interaction with pKal by ELISA, and hits were characterized using purified Fab fragments. Of these, one Fab (M0162-A04) was selected for affinity maturation given that it inhibited the proteolytic activity of both human and rat pKal with equal potency (*K*_*i*_^app^ ≈5 nm), while not binding prekallikrein (data not shown). A comparison of apparent inhibition constants (*K*_*i*_^app^) and HV-CDR3 amino acid sequences for the most potent inhibitors obtained from the affinity maturation campaign (Table 1) revealed that only a few amino acid substitutions were necessary to significantly improve potency. For instance, the introduction of a valine at position 5 (I103V) in HV-CDR3 alone improved the affinity almost 10-fold (*e.g.* the M0201-H06 Fab, *K*_*i*_^app^ = 0.56 nm), although the combination of T101I and A108E substitutions along with I103V (isolate M0199-A08) further improved the potency toward pKal (*K*_*i*_^app^ = 0.05 nm). Including rational mutagenesis (Table 1) and consideration of invariant residues post affinity maturation (*e.g.* Pro-104 and Arg-105), a common motif for the HV-CDR3 interaction with pKal is evident (shown as a modified SeqLogo in Table 1). Ultimately, the M0199-A08 variant was chosen to be reformatted for production as a germ line-optimized human IgG1 in CHO cells given its favorable potency, specificity, and protein expression/stability properties, and it is hereafter referred to as DX-2930.

**TABLE 1:**
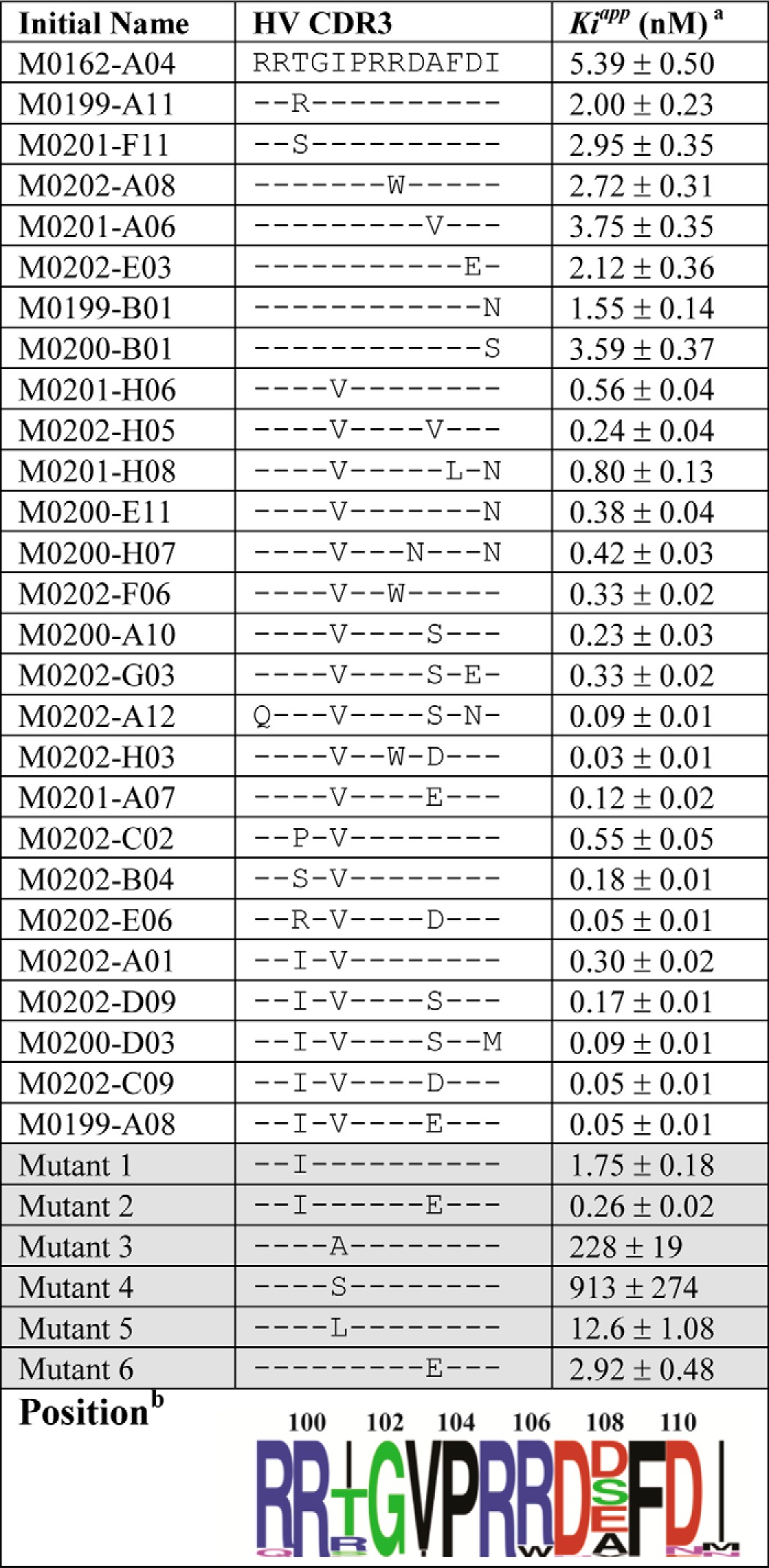
**Summary of HV-CDR3 affinity maturation**

*^a^ K*_*i*_^app^ determined for full-length pKal by potency studies like those shown in Fig. *2A*.

*^b^* Residue positions were specific for DX-2930. Sequence logo is shown for the affinity maturation output and weighting isolates for their capacity to inhibit pKal (higher weight to lower *K_i_*).

##### DX-2930 Is a Potent Inhibitor of pKal

DX-2930 retains its potent *in vitro* inhibition of pKal as a full-length human IgG1 (Fig. 2*A*). The observed linear increase in *K*_*i*_^app^ values with increasing substrate concentrations is consistent with a competitive inhibition mechanism for DX-2930 (Fig. 2*A*, *inset*). Using Equation 2, the inhibition constant *K_i_* was calculated at each substrate concentration and averaged (*K_i_* = 0.120 ± 0.005 nm) (Fig. 2*A*). DX-2930 also inhibited purified pKal from mouse (*K*_*i*_^app^ = 0.17 nm), rat (*K*_*i*_^app^ = 0.17 nm), rabbit (*K*_*i*_^app^ = 14.2 nm), and cynomolgus monkey (*K*_*i*_^app^ = 0.07 nm) (data not shown).

In plasma, a major fraction of circulating pKal is bound to HMWK via sites distal from both the pKal catalytic domain and the bradykinin-containing domain of HMWK (1). Therefore, we also tested whether DX-2930 inhibits pKal complexed with 2-chain HMWK, a form of HMWK that lacks bradykinin (Fig. 2*A*). The observed dissociation constant (*K_D_*) for the interaction of pKal with sensor chip-immobilized 2-chain HMWK was determined by surface plasmon resonance experiments (Fig. 2*B*, *K_D_* = 72 nm). Similar, or even higher affinity, dissociation constants (15 nm) have been previously reported for the interaction of pKal with 1-chain HMWK (47). Thus, at the physiologic HMWK concentration used in Fig. 2*A* (600 nm) (48) the catalytic amount of pKal present is essentially fully bound to HMWK. As shown in Fig. 2*A*, DX-2930 inhibits both free and HMWK-bound pKal with equivalent *K*_*i*_^app^ values. Consistent with the formation of a saturated pKal·HMWK complex, we obtained similar *K*_*i*_^app^ values at higher HMWK concentrations (4.7 μm) (data not shown).

DX-2930 also effectively targets the pKal·HMWK complex when bound to endothelial cells (Fig. 2*C*). Binding of pKal to HMWK has been previously shown to lead to the association of the resulting complex with endothelial cells (1). Specifically, ELISA experiments verified that biotinylated DX-2930 only interacted with HUVEC cells pre-loaded with pKal complexed with cell-bound HMWK. In contrast, C1-INH interacted poorly with similarly treated cells.

In addition to the above experiments that utilize a small peptide substrate, we further show that DX-2930 is a potent inhibitor of the pKal-mediated proteolysis of single-chain HMWK (its endogenous protein substrate). Addition of DX-2930 to pKal samples *in vitro* prevented the proteolysis of HMWK and the consequent release of bradykinin with an IC_50_ = 1.3 nm, as measured by bradykinin-specific ELISAs (Fig. 2*D*). This observed IC_50_ value is higher than the *K*_*i*_^app^ value for DX-2930, which may be attributed to technical challenges with using an ELISA for enzyme kinetic measurements.

##### DX-2930 Only Inhibits Active pKal

Several findings established that DX-2930 specifically targets activated pKal, and in particular the active site of the enzyme. First, DX-2930 did not inhibit any of the 20 different serine proteases listed under “Experimental Procedures” (including the 68% identical FXIa catalytic domain) at a final concentration of 1 μm DX-2930. Second, SPR experiments (Fig. 2*E*) demonstrated that DX-2930 binds active pKal, but binding is markedly diminished to an equivalent amount of prekallikrein or to pKal that has been inactivated with a small molecule, active site-directed inhibitor (AEBSF). This latter finding suggests that the antibody interacts with the pKal active site given that AEBSF covalently modifies the active site serine (49). Importantly, SDS-PAGE experiments that analyze DX-2930 samples preincubated with and without an equimolar concentration (2 μm) of pKal demonstrate that the antibody is not cleaved (Fig. 2*F*).

##### Structure of the DX-2930·pKal Complex

**FIGURE 3.**
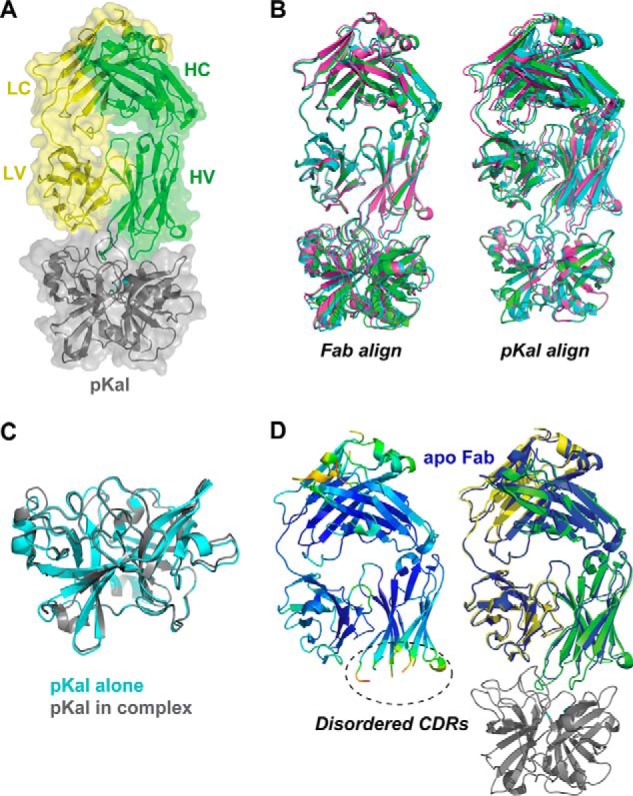
**X-ray crystallographic analysis of the pKal catalytic domain complexed with the DX-2930 Fab.** *A,* ribbon structure of the pKal·DX-2930 Fab complex (PDB 4OGX) with transparent surface rendering. pKal ribbon is colored in *gray* with *cyan* marking the catalytic triad residues, and the DX-2930 Fab is in *green* (heavy chain) and *yellow* (light chain). *B,* all three resolved DX-2930·pKal complex structures from this study overlaid in ribbon (orientation as in *A*) with alignment on the Fab domain or pKal catalytic domain. Complex from PDB 4OGX is colored in *magenta*, and the two distinct complexes from PDB 4OGY are in *cyan* and *green. C,* overlay of pKal catalytic domain structure alone (*cyan*, PDB 1ANY) with the pKal catalytic domain crystallized in complex with DX-2930 (*gray*, from PDB 4OGX) reveals minimal changes in the bound/unbound forms (r.m.s.d. 0.36 Å). *D, left,* crystal structure of the unbound DX-2930 Fab (PDB 4PUB) in ribbon with color reflecting B-factor values reveals disordered heavy chain CDR loops but an intact light chain CDR2. *Right,* overlay of the apo-DX-2930 Fab (now all in *blue*) overlaid on the pKal·DX-2930 Fab complex structure (colored as in *A*).

To understand the interaction between DX-2930 and pKal with atomic resolution, we crystallized the Fab portion of DX-2930 both alone and in a complex with a deglycosylated variant of the human pKal catalytic domain (35). Diffraction data for Fab-only crystals (collected to 1.75 Å) and the pKal·DX-2930 Fab complex (to 2.1 and 2.4 Å for different crystal forms) was used to solve the structures by molecular replacement methods that employed the coordinates of both a generalized Fab framework without CDR loops and the pKal catalytic domain structure PDB 2ANY as the search models. Subsequent building/refinement resulted in the final complex and apo-Fab structures presented in Fig. 3, with diffraction and refinement statistics presented in Table 2. Overall, the three final complex structures (one in the asymmetric unit of PDB 4OGX and two in the asymmetric unit of PDB 4OGY) are highly similar, with small adjustments in the interface such that alignments centered on either the Fab or pKal yield lower deviations for the respective chains (Fig. 3*B*). Both the pKal catalytic domain (r.m.s.d. 0.36 Å, Fig. 3*C*) and DX-2930 Fab (r.m.s.d. 1.09 Å, Fig. 3*D*) that are present in the complex deviate little from their individual apo structures. However, DX-2930 heavy chain CDR loops do not have resolvable density in the apo structure.

**TABLE 2:** **X-ray crystallographic collection and refinement statistics**

PDB ID	4OGX	4OGY	4PUB
Crystal ID	242118h7	242121d10	243341c6
Precipitant	0.1 m imidazole, pH 6.5, 30% MPD*^a^*, 0.2 m (NH_4_)_2_SO_4_, 10% PEG 3350	0.1 m BisTris*^a^*, pH 6.5, 20% PEG 5000 MME	0.09% BisTris, pH 5.5, 22.5% PEG 3500, 3% DMSO
Cryoprotectant	None	20% ethylene glycol	None
Crystallization Temperature	16 °C	16 °C	16 °C
Beam line	Rigaku FR-E+ SuperBright/Saturn 944+ CCD	Rigaku FR-E+ SuperBright/Saturn 944+ CCD	APS LS-CAT 21 ID-F
Space group	*P*2_1_2_1_2_1_	*P*2_1_	*P*2_1_2_1_2_1_
Unit cell	*a* = 109.6, *b* = 171.5, *c* = 42.32 Å	*a* = 82.12, *b* = 113.8, *c* = 89.05 Å	*a* = 45.93, *b* = 60.54, *c* = 150.49 Å
	α = β = γ = 90°	α = γ = 90°, β = 94.70°	α = β = γ = 90°
Solvent content	56%	54%	44%
*V_m_*	2.8 Å^3^/Da	2.67 Å^3^/Da	2.2 Å^3^/Da
Resolution	50-2.4 Å (2.45-2.4 Å)*^b^*	50-2.1 Å (2.14-2.10 Å)	50-1.75 Å (1.79–1.75 Å)
*I*/σ	15.7 (2.9)	22.6 (5.8)	22.4 (3.8)
Completeness	99.9% (99.6%)	99.8% (99.8%)	99.1% (99.8%)
*R*_merge_	0.112 (0.477)	0.076 (0.364)	0.051 (0.471)
Multiplicity	6.7 (4.0)	7.3 (6.1)	6.1 (6.1)
Reflections	32,218 (2323)	94,897 (6934)	42,967 (3147)
Mosaicity	0.6	0.3	0.3

**Refinement**			
*R*	0.188 (0.241)	0.185 (0.202)	0.174 (0.242)
*R*_free_	0.236 (0.296)	0.221 (0.244)	0.214 (0.289)
Validation*^c^*			
Ramachandran favored	96.8%	97.5%	97.8%
Ramachandran outliers	0.0%	0.0%	0.2%
Rotamer outliers	1.8%	0.8%	1.4%
Clash score	5.68 (99th percentile)	2.71 (99th percentile)	5.35 (94th percentile)
Molprobity score	1.71 (98th percentile)	1.17 (100th percentile)	1.45 (95th percentile)

*^a^* BisTris is 2-[bis(2-hydroxyethyl)amino]-2-(hydroxymethyl)propane-1,3-diol and MPD is 2-methyl-2,4-pentanediol.

*^b^* Statistics for highest resolution shell data are shown in parentheses throughout the table.

*^c^* Validation statistics were obtained from Molprobity (79).

**FIGURE 4.**
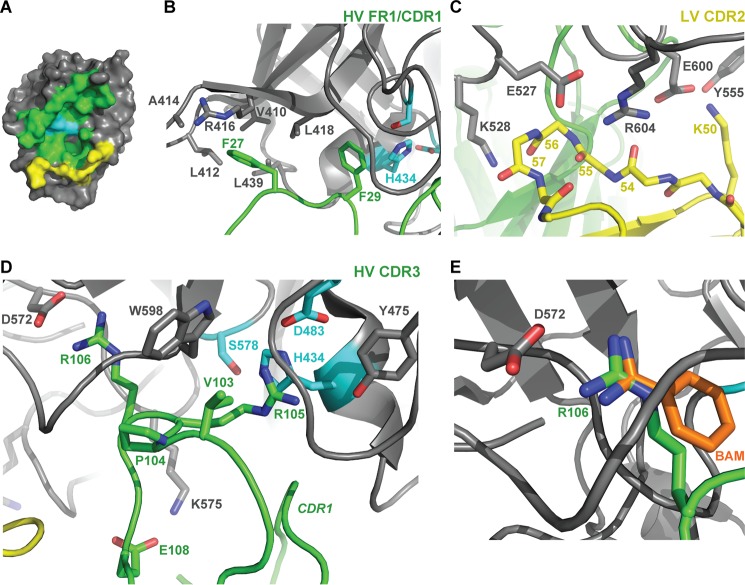
**Structural analysis of DX-2930 CDR interactions with the pKal catalytic domain.** *A,* surface rendering of the pKal catalytic domain highlighting complex interface residues, with heavy chain-interacting residues in *green* and light chain-interacting ones in *yellow*. Detailed views of critical side-chain interactions between pKal and the DX-2930 Fab residues are shown as follows: *B,* heavy chain FR1 and CDR1 (HV FR1/CDR1); *C,* light chain CDR2 (LV CDR2); and *D,* heavy chain CDR3 (HV CDR3). *E,* overlay of the benzamidine molecule from PDB 2ANY reveals a bidentate interaction with pKal residue Asp-572 (chymotrypsin Asp-195) in a manner analogous to Arg-106 of the DX-2930 Fab.

Inspection of the complex structure reveals the reason why DX-2930 prevents substrate proteolysis by pKal. Namely, the Fab interface covers ∼2400 Å^2^ of surface and completely obstructs the active site of pKal (Fig. 4*A*). Direct contributions to this interface are made by Fab CDR and framework (FR) residues from both the heavy chain (HV-FR1, HV-CDR1, and HV-CDR3) and the light chain (LV-CDR2). Hydrophobic interactions of HV-FR1 residues, particularly Phe-27 and Phe-29, dominate the interface of this motif with pKal residues Val-410, Leu-412, Arg-416, Leu-418, Leu-439, Lys-575, and the His-434 of the pKal catalytic triad (Fig. 4*B*; prekallikrein numbering is used). For reference, the pKal residues His-434, Asp-483, and Ser-578 are equivalent to chymotrypsin catalytic triad residues His-57, Asp-102, and Ser-195, respectively. Moreover, His-31 of CDR1 forms a hydrogen bond with the backbone carbonyl of pKal residue Asp-437, and it consequently flips the Asp-437–Gly-438 peptide bond relative to the apo structure of the pKal catalytic domain. On the opposite side of the complex interface, Lys-50 of LV-CDR2 makes a side chain-mediated interaction with pKal residues Tyr-555 and Glu-600 (Fig. 4*C*). This interface is also supported by several backbone-mediated hydrogen bonds made by pKal residues Glu-527 (contacting amide nitrogen of Gly-57), Lys-528 (contacting carbonyl oxygen of Ser-56), and Arg-604 (interacting with carbonyl oxygen atoms of Leu-54 and Glu-55).

The most intimate interactions between the DX-2930 Fab and pKal catalytic domain are mediated by the same heavy chain CDR3 residues that led to affinity improvements (Table 1). The CDR3 loop extends deeply into the active site of pKal (Fig. 4*D*) and interacts with both the substrate binding region and the catalytic triad. Arg-106 anchors the loop, burying into the canonical S1 binding pocket and forming a bidentate bonding interaction with pKal D572, much like the broad serine protease inhibitor benzamidine (Fig. 4, *D* and *E*). This interaction is preceded on the N-terminal side of the CDR by contacts of both Pro-104 and Arg-105, which abuts the catalytic triad, and directly bonds with Asp-483, with the S3 and S2 substrate binding pockets of pKal, respectively. Val-103 appears to have enough hydrophobic character to interact with pKal Tyr-555 and Trp-598 but not so large that it disrupts a nearby loop in pKal (residues 478–480) that further interacts/stabilizes both HV CDR1 and CDR3. Finally, Glu-108, which is predominantly acidic/polar in the most inhibitory affinity maturation variants, contacts Lys-575 of pKal in an interaction that is likely to be a significant driver of DX-2930 specificity with respect to other serine proteases, most of which have a Gln or Glu at this position (35). Although not directly involved in pKal interactions, the CDR3 residues ^99^RRIG, Asp-107, and ^109^FDI that were also included in the affinity maturation rounds make Fab intramolecular contacts that stabilize the CDR3 loop conformation and also likely contribute to the overall folding stability of the Fab.

Taken together, the composition and extended length of the HV CDR3 allows the intimate contact of this loop with the pKal active site, while being short enough to steer the potentially labile peptide backbone of this loop away from the proteolytic residues of pKal. Of note, the complex structure is consistent with a proposed competitive inhibition mechanism given that binding of this antibody to pKal will exclude even small peptide substrates from the pKal active site.

##### Pharmacokinetic and Pharmacodynamic Analysis of DX-2930

**FIGURE 5.**
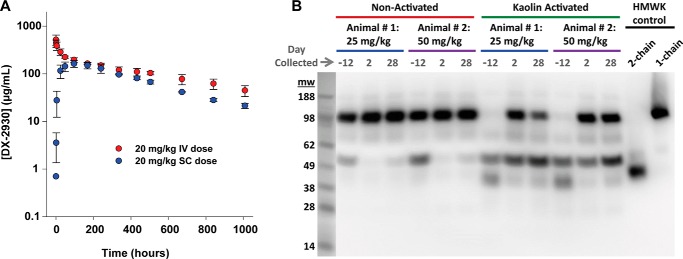
**Pharmacokinetic and pharmacodynamic properties of DX-2930 in cynomolgus monkey.** *A,* 20 mg/kg DX-2930 was dosed by either intraperitoneal (*IP*) or subcutaneous (*SC*) injection. Citrated plasma was collected at indicated times (hours) post-dose, and the concentration of DX-2930 was measured by a quantitative ELISA utilizing an antibody specific for DX-2930 (see “Experimental Procedures”). The s.c. dosing results in a maximal DX-2930 concentration reached by 96 h and a prolonged serum half-life of ∼12.5 days. Noncompartmental pharmacokinetic analyses yield the parameters shown in Table 3. *Error bars* shown are the standard deviation between replicates (*n* = 3). *B,* inhibition of pKal-mediated HMWK cleavage by DX-2930 in activated plasma. The KKS in plasma collected from cynomolgus monkeys subcutaneously treated with 25 mg/kg or 50 mg/kg DX-2930 was activated by kaolin addition and samples analyzed by Western blots detecting intact 1-chain or proteolyzed 2-chain HMWK. A light image of included molecular mass standards (in kDa) is shown adjacent to the Western blot image. Purified human 1- and 2-chain HMWK at 5 nm were included as controls. Addition of DX-2930 reduces HMWK proteolysis relative to nondosed samples (day −12) even when sampled 28 days post-injection.

To develop a long term prophylactic inhibitor of pKal, it was necessary to demonstrate a sustained bioactivity of DX-2930 *in vivo*. Toward this end, the pharmacokinetic profile of DX-2930 injected both s.c. and i.v. into cynomolgus monkeys at 20 mg/kg was examined, revealing a high bioavailability (66%) and sustained half-life of ∼12.5 and 19.3 days, respectively (Fig. 5*A* and Table 3). The injected dose of DX-2930 also retains bioactivity throughout its residence in the circulation. Specifically, plasma samples from monkeys that were dosed with or without s.c. DX-2930 were treated *ex vivo* with kaolin to activate the contact system, and the proteolytic activity of the resulting pKal was assessed by monitoring the presence or absence of cleaved 2-chain HMWK by Western blot analysis (Fig. 5*B*). DX-2930 inhibited the pKal-mediated proteolysis of HMWK even at 28 days following s.c. dosing, a finding consistent with the prolonged serum half-life of DX-2930.

**TABLE 3:** **Pharmacokinetic parameters of DX-2930 in cynomolgus monkeys**

Group	Route	*C*_max_	AUC_last_	CL	*Vss*	*t*½
		μ*g/ml*	*h* × μ*g/ml*	*ml/h/kg*	*ml/kg*	*h*
1	i.v.	519.7 (133.1)	116,447.4*^a^* (16463.2)	0.14 (0.03)	86.3 (5.6)	463.8 (68.9)
2	s.c.	162.8 (26.3)	77,024.8*^a^* (6252.0)	0.23 (0.02)	NA	301.1 (5.4)

*^a^* The percent bioavailability when administered by the s.c. route was approximately 66% (s.c. AUC_last_/i.v. AUC_last_), where AUC_last_ is area under the curve from time 0 to the time of last quantifiable concentration; Vss is apparent volume of distribution at steady state; CL is clearance. Values in parentheses are standard errors of the mean as determined by WinNonlin noncompartmental analyses (Pharsight).

##### DX-2930 Treatment Reduces Carrageenan-induced Paw Edema in Rats

**FIGURE 6.**
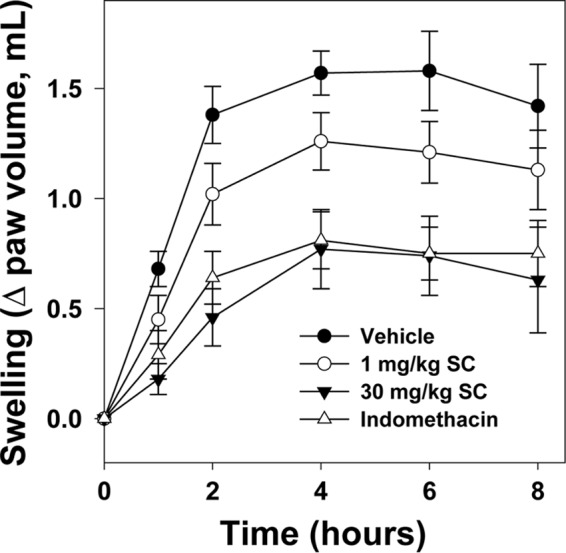
**DX-2930 inhibits carrageenan-induced paw edema in the rat.** DX-2930 was administered via a s.c. injection 24 h prior to carrageenan injection into the rat hind paw, although the vehicle (Formulation Buffer) and indomethacin (5 mg/kg) controls were administered by i.p. injection 30 min prior to carrageenan treatment. Paw volume was measured at the indicated time points following carrageenan injection and averaged for each treatment group (*n* = 10). Error calculated using the standard deviation.

To begin addressing the *in vivo* efficacy of DX-2930, we investigated the ability of DX-2930 to diminish carrageenan-induced paw edema (CPE) in rats. CPE is not a disease model of HAE but is a commonly used model of general inflammation that involves several mediators including pro-inflammatory cytokines, histamine, 5-hydroxytryptamine, and prostaglandins, in addition to kinins (50, 51). Rats were pretreated with s.c. DX-2930 24 h prior to the injection of 1% carrageenan into the right hind paw to induce edema, and dose-dependent reductions in swelling were recorded (Fig. 6). DX-2930 (30 mg/kg) reduced paw edema at 8 h by 55.6% compared to vehicle-treated rats. The COX inhibitor indomethacin was used as a positive control and reduced paw edema at 8 h by 47.2% compared with vehicle. Full inhibition is not reached by either inhibitor as expected given that multiple parallel inflammatory pathways cause CPE. However, the significant reduction in CPE with both DX-2930 and indomethacin treatment does demonstrate the inhibition of kinin-mediated prostaglandin biosynthesis in this model. Furthermore, the efficacy of DX-2930 24 h after subcutaneous dosing demonstrates the potential for this antibody in prophylactic KKS inhibition.

## DISCUSSION

Dysregulated contact system activation is central to the pathophysiology of HAE, and HAE attacks can be effectively treated with inhibitors that either prevent pKal-mediated proteolytic production of bradykinin or antagonize the B2 receptor. We used phage display to select and develop a potent, specific, and long-lived antibody inhibitor of pKal, DX-2930. X-ray crystallographic analysis of the DX-2930 Fab region bound to pKal details the molecular basis of our discovery, demonstrating that the antibody binds and occludes the substrate-binding active site region of the protease.

**FIGURE 7.**
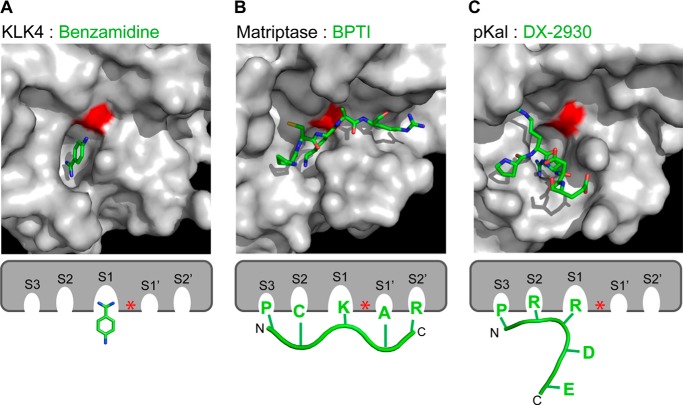
**Comparison of inhibitor interactions with serine protease active site pockets.** Surface rendering of serine proteases are shown docked with stick models of their respective ligands just above the schematic models of these interactions with canonical protease docking sites highlighted. *A,* small molecule benzamidine docks into the deep S1 binding pocket of KLK4 (PDB 2BDG). *B,* peptide sequence from the small protein BPTI binds matriptase in a “substrate-like” mode (PDB 1EAW). *C,* part of the HV-CDR3 of DX-2930 binds the S1-S3 sites of pKal and then abruptly turns away from the catalytic serine. *N* and *C* refer to N- and C-terminal ends of docked schematic peptides. Inhibitor stick color scheme by atom: *blue,* nitrogen; *red,* oxygen; *green,* carbon. *Red* in surface representation and *red asterisk* in schematic representation denote the catalytic serine residue.

The DX-2930 mechanism of inhibition is similar to that employed by other well described serine protease inhibitors. For instance, amino acid Arg-106 of DX-2930 HV-CDR3 buries deeply into the canonical S1 substrate binding pocket in a manner similar to both benzamidine (Fig. 4, *D* and *E,* and Fig. 7*A*) and the lysine side chain of the broad specificity inhibitor BPTI/aprotinin (Fig. 7*B*). With respect to reported antibody inhibitors of serine proteases, Craik and co-workers (52) characterized three antibodies that bind matriptase (MT-SP1) whereby the interacting HV-CDR3 residues align in a C-terminal to N-terminal orientation rather than the substrate-like N-terminal to C-terminal orientation (*e.g.* BPTI in Fig. 7*B*). This reverse orientation allows the antibodies to bind the scission machinery of an active protease without resulting in their proteolysis. In contrast, DX-2930 binds in the substrate-like N-terminal to C-terminal orientation in the S1-S3 subsites, even to the extent that it chemically and structurally mimics the P3 proline and P1 arginine present in the natural pKal substrate, HMWK. However, the orientation of the HV-CDR3 loop in DX-2930 prevents interactions with subsites S1′-S3′, and thereby it prevents its cleavage by the pKal catalytic serine (Fig. 7*C*). Importantly from the perspective of an antibody therapeutic, we demonstrate that DX-2930 is indeed not proteolyzed by pKal (Fig. 2*F*) despite such an intimate complex formation.

The crystal structure of prekallikrein is not available, but the likely structural homology to the related proenzyme form of kallikrein 6, whose crystal structure has been defined (53), suggests that the S1 pocket (and other active site residue conformations) would only be present in the activated pKal form and not in prekallikrein. Thus, given the significant contact of DX-2930 HV-CDR3 residues with the pKal active site, it is not surprising that our SPR binding studies (Fig. 2*E*) demonstrate that DX-2930 does not bind prekallikrein.

A lack of prekallikrein binding was an intentional primary selection criterion to potentially reduce the therapeutic levels of DX-2930 necessary to inhibit pKal activity. Prekallikrein is present in plasma at a concentration of ∼500 nm, and we reasoned that binding to prekallikrein would necessitate drug levels at equivalent concentrations. In contrast, a potent antibody inhibitor that bound only the active form of the enzyme could be dosed to levels that match the amount of pKal generated in disease. pKal levels between 30 and 110 nm have been estimated in HAE plasma, and an effective dose of ecallantide for the treatment of acute attacks of HAE has a C_max_ of ∼80 nm (586 ng/ml) (54, 55). Therapeutic pKal inhibition has the potential to attenuate kinin production, as well as to limit the formation of active pKal generated by the cyclical pKal-mediated conversion of FXII to FXIIa. Therefore, it is possible that prophylactic inhibition of pKal activity may require a lower dose of inhibitor than that needed to acutely treat an HAE attack. However, future clinical studies in HAE will help elucidate the DX-2930 dose necessary for successful long term attack prophylaxis.

Certain properties of DX-2930 may support its use as a novel reagent to investigate contact system activation in biological systems or as a potential therapy for the treatment of pKal-mediated diseases, such as HAE. First, the specificity of DX-2930 toward binding activated pKal, rather than prekallikrein or any other tested serine protease suggests that it could prove to be a useful reagent to detect biological sites where active pKal is generated. Second, DX-2930 can target free soluble pKal, as well as pKal complexed with HMWK that may be further associated with endothelial cells (Fig. 2, *A–C*). In contrast, pKal is resistant to inhibition by endogenous inhibitors C1-INH and α2-macroglobulin (α2M) when it is bound to HMWK on endothelial cells, a property it shares with other cell-bound proteases such as plasmin and urokinase-type plasminogen activator (56, 57). Such observations support the concept that HAE attack localization may be partially attributed to endothelial cell-bound pKal. Third, our studies in non-human primates demonstrate that DX-2930 has a long pharmacokinetic half-life (12.5 days in non-human primates, Fig. 5*A* and Table 3). Our recent completion of a phase 1A clinical study further reveals that DX-2930 has an almost 3-week half-life in healthy human individuals (58). Finally, DX-2930 exhibits striking specificity for pKal, which differs from the endogenous C1-INH, which has likely evolved to target numerous serine proteases and has been reported to inhibit 12 of the 20 human serine proteases that were tested in this study (59–69). Taken together, high therapeutic doses of C1-INH are likely required for the treatment of HAE to offset its limited potency and broad specificity and may account for the occurrence of breakthrough attacks with current prophylactic approaches (70).

Contact system activation is observed in a variety of diseases associated with the hallmarks of inflammation (heat, redness, pain, and swelling) (1), implying that the activation and regulation of the contact system is tightly controlled in normal physiology. Here, we demonstrate using DX-2930-treated rats that plasma kallikrein is a mediator in CPE. Although not a specific disease model of HAE, bradykinin is one of several mediators in CPE (51), and the observed efficacy of DX-2930 in this model supports its on-target *in vivo* bioactivity. As a highly specific pKal inhibitor with *in vivo* efficacy, DX-2930 can be used to investigate the therapeutic benefit of pKal inhibition in other diseases reported to have elevated contact system activation. The role of the plasma KKS system in thrombosis has been particularly intriguing given that the targeted disruption of contact system proteins appears to reduce thrombus formation without increasing bleeding in animal models of venous and arterial thrombosis (71–77).

Interestingly, for such a conserved biological system, a genetic deficiency in prekallikrein or other contact system proteins (FXII and HMWK) is not associated with disease, including risk for bleeding, which suggests that chronic pKal inhibition could be well tolerated (78). Combining its potency with expected long half-life in patient circulation, DX-2930 may prove to be an effective prophylactic treatment of chronic diseases mediated by contact system activation, including HAE.

## Footnotes

2 The abbreviations used are:

FXII: factor XII
AEBSF: 4-(2-aminoethyl) benzenesulfonyl fluoride hydrochloride
C1-INH: C1 Inhibitor
CDR: complementarity determining region
CPE: carrageenan-induced paw edema
Fab: fragment antigen binding domain
FXIIa: activated FXII
HAE-C1INH: hereditary angioedema with C1-inhibitor deficiency
HMWK: high molecular weight kininogen
HV: antibody heavy chain variable domain
LV: antibody light chain variable domain
KKS: plasma kallikrein-kinin system
pKal: activated plasma kallikrein
SPR: surface plasmon resonance
cv: column volume
PDB: Protein Data Bank
r.m.s.d.: root mean square deviation
AMC: amido-4-methylcoumarin
RU: response unit
Z: benzyloxycarbonyl
CDR: complementary determining region
FR: framework.

## References

[1] Colman R. W. Schmaier A. H. (1997) Contact system: a vascular biology modulator with anticoagulant, profibrinolytic, antiadhesive, and proinflammatory attributes. Blood 90, 3819–3843 9354649

[2] Leeb-Lundberg L. M. Marceau F. Müller-Esterl W. Pettibone D. J. Zuraw B. L. (2005) International union of pharmacology. XLV. Classification of the kinin receptor family: from molecular mechanisms to pathophysiological consequences. Pharmacol. Rev. 57, 27–77 15734727 10.1124/pr.57.1.2

[3] Oschatz C. Maas C. Lecher B. Jansen T. Björkqvist J. Tradler T. Sedlmeier R. Burfeind P. Cichon S. Hammerschmidt S. Müller-Esterl W. Wuillemin W. A. Nilsson G. Renné T. (2011) Mast cells increase vascular permeability by heparin-initiated bradykinin formation *in vivo*. Immunity 34, 258–268 21349432 10.1016/j.immuni.2011.02.008

[4] Maas C. Govers-Riemslag J. W. Bouma B. Schiks B. Hazenberg B. P. Lokhorst H. M. Hammarström P. ten Cate H. de Groot P. G. Bouma B. N. Gebbink M. F. (2008) Misfolded proteins activate factor XII in humans, leading to kallikrein formation without initiating coagulation. J. Clin. Invest. 118, 3208–3218 18725990 10.1172/JCI35424 PMC2518075

[5] van der Meijden P. E. Munnix I. C. Auger J. M. Govers-Riemslag J. W. Cosemans J. M. Kuijpers M. J. Spronk H. M. Watson S. P. Renné T. Heemskerk J. W. (2009) Dual role of collagen in factor XII-dependent thrombus formation. Blood 114, 881–890 19372258 10.1182/blood-2008-07-171066

[6] Schousboe I. Nystrøm B. (2009) High molecular weight kininogen binds to laminin–characterization and kinetic analysis. FEBS J. 276, 5228–5238 19691495 10.1111/j.1742-4658.2009.07218.x

[7] White-Adams T. C. Berny M. A. Patel I. A. Tucker E. I. Gailani D. Gruber A. McCarty O. J. (2010) Laminin promotes coagulation and thrombus formation in a factor XII-dependent manner. J. Thromb. Haemost. 8, 1295–1301 20796202 10.1111/j.1538-7836.2010.03850.x PMC4367539

[8] Kannemeier C. Shibamiya A. Nakazawa F. Trusheim H. Ruppert C. Markart P. Song Y. Tzima E. Kennerknecht E. Niepmann M. von Bruehl M. L. Sedding D. Massberg S. Günther A. Engelmann B. Preissner K. T. (2007) Extracellular RNA constitutes a natural procoagulant cofactor in blood coagulation. Proc. Natl. Acad. Sci. U.S.A. 104, 6388–6393 17405864 10.1073/pnas.0608647104 PMC1851071

[9] Smith S. A. Mutch N. J. Baskar D. Rohloff P. Docampo R. Morrissey J. H. (2006) Polyphosphate modulates blood coagulation and fibrinolysis. Proc. Natl. Acad. Sci. U.S.A. 103, 903–908 16410357 10.1073/pnas.0507195103 PMC1347979

[10] Müller F. Mutch N. J. Schenk W. A. Smith S. A. Esterl L. Spronk H. M. Schmidbauer S. Gahl W. A. Morrissey J. H. Renné T. (2009) Platelet polyphosphates are proinflammatory and procoagulant mediators *in vivo*. Cell 139, 1143–1156 20005807 10.1016/j.cell.2009.11.001 PMC2796262

[11] Sanchez J. Elgue G. Larsson R. Nilsson B. Olsson P. (2008) Surface-adsorbed fibrinogen and fibrin may activate the contact activation system. Thromb. Res. 122, 257–263 18177925 10.1016/j.thromres.2007.11.008

[12] Tapper H. Herwald H. (2000) Modulation of hemostatic mechanisms in bacterial infectious diseases. Blood 96, 2329–2337 11001879

[13] Nielsen V. G. Steenwyk B. L. Gurley W. Q. (2006) Contact activation prolongs clot lysis time in human plasma: role of thrombin-activatable fibrinolysis inhibitor and Factor XIII. J. Heart Lung Transplant. 25, 1247–1252 17045938 10.1016/j.healun.2006.06.009

[14] Rahman M. M. Bhoola K. D. Elson C. J. Lemon M. Dieppe P. A. (1995) Identification and functional importance of plasma kallikrein in the synovial fluids of patients with rheumatoid, psoriatic, and osteoarthritis. Ann. Rheum. Dis. 54, 345–350 7794038 10.1136/ard.54.5.345 PMC1005592

[15] Stadnicki A. Gonciarz M. Niewiarowski T. J. Hartleb J. Rudnicki M. Merrell N. B. Dela Cadena R. A. Colman R. W. (1997) Activation of plasma contact and coagulation systems and neutrophils in the active phase of ulcerative colitis. Dig. Dis. Sci. 42, 2356–2366 9398817 10.1023/a:1018891323205

[16] Weiser P. Qian Y. Pan J. Zhou X. Lu H. Studelska D. R. Shih F. F. Zhang L. (2010) Activated contact system and abnormal glycosaminoglycans in lupus and other auto- and non-autoimmune diseases. Prog. Mol. Biol. Transl. Sci. 93, 443–472 20807656 10.1016/S1877-1173(10)93019-6

[17] Phipps J. A. Feener E. P. (2008) The kallikrein-kinin system in diabetic retinopathy: lessons for the kidney. Kidney Int. 73, 1114–1119 18272958 10.1038/ki.2008.9

[18] Clermont A. Chilcote T. J. Kita T. Liu J. Riva P. Sinha S. Feener E. P. (2011) Plasma kallikrein mediates retinal vascular dysfunction and induces retinal thickening in diabetic rats. Diabetes 60, 1590–1598 21444925 10.2337/db10-1260 PMC3292335

[19] Coppola R. Cristilli P. Cugno M. Ariëns R. A. Mari D. Mannucci P. M. (1996) Measurement of activated factor XII in health and disease. Blood Coagul. Fibrinolysis 7, 530–535 8874863 10.1097/00001721-199607000-00004

[20] Miller R. L. Verma P. S. Adams R. G. (1983) Studies of the kallikrein-kinin system in patients with sickle cell anemia. J. Natl. Med. Assoc. 75, 551–556 6603519 PMC2561580

[21] Bergamaschini L. Donarini C. Gobbo G. Parnetti L. Gallai V. (2001) Activation of complement and contact system in Alzheimer's disease. Mech. Ageing Dev. 122, 1971–1983 11589915 10.1016/s0047-6374(01)00311-6

[22] Makevnina L. G. Lomova I. P. Zubkov YuN. Semenyutin V. B. (1994) Kininogen consumption in cerebral circulation of humans during brain ischemia and postischemic reperfusion. Braz. J. Med. Biol. Res. 27, 1955–1963 7749387

[23] Cugno M. Salerno F. Mandelli M. Lorenzano E. Paonessa R. Agostoni A. (1995) Cleavage of high molecular weight kininogen in ascites and plasma of patients with cirrhosis. Thromb. Res. 78, 277–282 7631308 10.1016/0049-3848(95)00060-5

[24] Kaufman N. Page J. D. Pixley R. A. Schein R. Schmaier A. H. Colman R. W. (1991) α2-Macroglobulin-kallikrein complexes detect contact system activation in hereditary angioedema and human sepsis. Blood 77, 2660–2667 1710516

[25] Cugno M. Cicardi M. Bottasso B. Coppola R. Paonessa R. Mannucci P. M. Agostoni A. (1997) Activation of the coagulation cascade in C1-inhibitor deficiencies. Blood 89, 3213–3218 9129025

[26] Berrettini M. Lämmle B. White T. Heeb M. J. Schwarz H. P. Zuraw B. Curd J. Griffin J. H. (1986) Detection of *in vitro* and *in vivo* cleavage of high molecular weight kininogen in human plasma by immunoblotting with monoclonal antibodies. Blood 68, 455–462 3730610

[27] Bork K. Meng G. Staubach P. Hardt J. (2006) Hereditary angioedema: new findings concerning symptoms, affected organs, and course. Am. J. Med. 119, 267–274 16490473 10.1016/j.amjmed.2005.09.064

[28] Bork K. (2010) Diagnosis and treatment of hereditary angioedema with normal C1 inhibitor. Allergy Asthma Clin. Immunol. 6, 15 20667118 10.1186/1710-1492-6-15 PMC2919521

[29] Caliezi C. Wuillemin W. A. Zeerleder S. Redondo M. Eisele B. Hack C. E. (2000) C1-esterase inhibitor: an anti-inflammatory agent and its potential use in the treatment of diseases other than hereditary angioedema. Pharmacol. Rev. 52, 91–112 10699156

[30] Bork K. (2012) Current management options for hereditary angioedema. Curr. Allergy Asthma Rep. 12, 273–280 22729959 10.1007/s11882-012-0273-4

[31] Gandhi P. K. Gentry W. M. Bottorff M. B. (2012) Thrombotic events associated with C1 esterase inhibitor products in patients with hereditary angioedema: investigation from the United States Food and Drug Administration adverse event reporting system database. Pharmacotherapy 32, 902–909 23033229 10.1002/j.1875-9114.2012.01126

[32] Bork K. Bygum A. Hardt J. (2008) Benefits and risks of danazol in hereditary angioedema: a long-term survey of 118 patients. Ann. Allergy Asthma Immunol. 100, 153–161 18320917 10.1016/S1081-1206(10)60424-3

[33] Banerji A. Sloane D. E. Sheffer A. L. (2008) Hereditary angioedema: a current state-of-the-art review, V: attenuated androgens for the treatment of hereditary angioedema. Ann. Allergy Asthma Immunol. 100, S19–S22 18220148 10.1016/s1081-1206(10)60582-0

[34] Zuraw B. L. Busse P. J. White M. Jacobs J. Lumry W. Baker J. Craig T. Grant J. A. Hurewitz D. Bielory L. Cartwright W. E. Koleilat M. Ryan W. Schaefer O. Manning M. Patel P. Bernstein J. A. Friedman R. A. Wilkinson R. Tanner D. Kohler G. Gunther G. Levy R. McClellan J. Redhead J. Guss D. Heyman E. Blumenstein B. A. Kalfus I. Frank M. M. (2010) Nanofiltered C1 inhibitor concentrate for treatment of hereditary angioedema. N. Engl. J. Med. 363, 513–522 20818886 10.1056/NEJMoa0805538

[35] Tang J. Yu C. L. Williams S. R. Springman E. Jeffery D. Sprengeler P. A. Estevez A. Sampang J. Shrader W. Spencer J. Young W. McGrath M. Katz B. A. (2005) Expression, crystallization, and three-dimensional structure of the catalytic domain of human plasma kallikrein. J. Biol. Chem. 280, 41077–41089 16199530 10.1074/jbc.M506766200

[36] Hekim C. Leinonen J. Närvänen A. Koistinen H. Zhu L. Koivunen E. Väisänen V. Stenman U. H. (2006) Novel peptide inhibitors of human kallikrein 2. J. Biol. Chem. 281, 12555–12560 16527822 10.1074/jbc.M600014200

[37] Hoet R. M. Cohen E. H. Kent R. B. Rookey K. Schoonbroodt S. Hogan S. Rem L. Frans N. Daukandt M. Pieters H. van Hegelsom R. Neer N. C. Nastri H. G. Rondon I. J. Leeds J. A. Hufton S. E. Huang L. Kashin I. Devlin M. Kuang G. Steukers M. Viswanathan M. Nixon A. E. Sexton D. J. Hoogenboom H. R. Ladner R. C. (2005) Generation of high-affinity human antibodies by combining donor-derived and synthetic complementarity-determining-region diversity. Nat. Biotechnol. 23, 344–348 15723048 10.1038/nbt1067

[38] Wassaf D. Kuang G. Kopacz K. Wu Q. L. Nguyen Q. Toews M. Cosic J. Jacques J. Wiltshire S. Lambert J. Pazmany C. C. Hogan S. Ladner R. C. Nixon A. E. Sexton D. J. (2006) High-throughput affinity ranking of antibodies using surface plasmon resonance microarrays. Anal. Biochem. 351, 241–253 16510109 10.1016/j.ab.2006.01.043

[39] Jostock T. Vanhove M. Brepoels E. Van Gool R. Daukandt M. Wehnert A. Van Hegelsom R. Dransfield D. Sexton D. Devlin M. Ley A. Hoogenboom H. Müllberg J. (2004) Rapid generation of functional human IgG antibodies derived from Fab-on-phage display libraries. J. Immunol. Methods. 289, 65–80 15251413 10.1016/j.jim.2004.03.014

[40] Sexton D. J. Chen T. Martik D. Kuzmic P. Kuang G. Chen J. Nixon A. E. Zuraw B. L. Forteza R. M. Abraham W. M. Wood C. R. (2009) Specific inhibition of tissue kallikrein 1 with a human monoclonal antibody reveals a potential role in airway diseases. Biochem. J. 422, 383–392 19527222 10.1042/BJ20090010

[41] Cha S. (1975) Tight-binding inhibitors. I. Kinetic behavior. Biochem. Pharmacol. 24, 2177–2185 1212266 10.1016/0006-2952(75)90050-7

[42] Morrison J. F. (1969) Kinetics of the reversible inhibition of enzyme-catalysed reactions by tight-binding inhibitors. Biochim. Biophys. Acta 185, 269–286 4980133 10.1016/0005-2744(69)90420-3

[43] Kuzmic P. Elrod K. C. Cregar L. M. Sideris S. Rai R. Janc J. W. (2000) High-throughput screening of enzyme inhibitors: Simultaneous determination of tight-binding inhibition constants and enzyme concentration. Anal. Biochem. 286, 45–50 11038272 10.1006/abio.2000.4685

[44] Emsley P. Cowtan K. (2004) Coot: model-building tools for molecular graphics. Acta Crystallogr. D. Biol. Crystallogr. 60, 2126–2132 15572765 10.1107/S0907444904019158

[45] Collaborative Computational Project No. 4 (1994) The CCP4 suite: programs for protein crystallography. Acta Crystallogr. D. Biol. Crystallogr. 50, 760–763 15299374 10.1107/S0907444994003112

[46] Murshudov G. N. Vagin A. A. Dodson E. J. (1997) Refinement of macromolecular structures by the maximum-likelihood method. Acta Crystallogr. D. Biol. Crystallogr. 53, 240–255 15299926 10.1107/S0907444996012255

[47] Bock P. E. Shore J. D. Tans G. Griffin J. H. (1985) Protein-protein interactions in contact activation of blood coagulation. Binding of high molecular weight kininogen and the 5-(iodoacetamido) fluorescein-labeled kininogen light chain to prekallikrein, kallikrein, and theseparated kallikrein heavy and light chains. J. Biol. Chem. 260, 12434–12443 3850090

[48] Silverberg M. Reddigari S. R. Kaplan A. P. (1995) in Blood: Principles and Practice of Hematology (Handin R. T. Lux S. E. Stossel T. P., eds) pp. 1127–1150, Lippincott Williams & Wilkins, Philadelphia

[49] Markwardt F. Drawert J. Walsmann P. (1974) Synthetic low molecular weight inhibitors of serum kallikrein. Biochem. Pharmacol. 23, 2247–2256 4859519 10.1016/0006-2952(74)90554-1

[50] Wirth K. J. Alpermann H. G. Satoh R. Inazu M. (1992) The bradykinin antagonist Hoe 140 inhibits carrageenan- and thermically induced paw oedema in rats. Agents Actions 38, 428–431 1462876

[51] Morris C. J. (2003) Carrageenan-induced paw edema in the rat and mouse. Methods Mol. Biol. 225, 115–121 12769480 10.1385/1-59259-374-7:115

[52] Schneider E. L. Lee M. S. Baharuddin A. Goetz D. H. Farady C. J. Ward M. Wang C. I. Craik C. S. (2012) A reverse binding motif that contributes to specific protease inhibition by antibodies. J. Mol. Biol. 415, 699–715 22154938 10.1016/j.jmb.2011.11.036 PMC3268006

[53] Gomis-Rüth F. X. Bayés A. Sotiropoulou G. Pampalakis G. Tsetsenis T. Villegas V. Avilés F. X. Coll M. (2002) The structure of human prokallikrein 6 reveals a novel activation mechanism for the kallikrein family. J. Biol. Chem. 277, 27273–27281 12016211 10.1074/jbc.M201534200

[54] Stolz L. E. Sheffer A. L. (2012) Prospective, double-blind, placebo-controlled trials of ecallantide for acute attacks of hereditary angioedema. Expert Rev. Clin. Immunol. 8, 25–32 22149337 10.1586/eci.11.81

[55] Bernstein J. A. Qazi M. (2010) Ecallantide: its pharmacology, pharmacokinetics, clinical efficacy and tolerability. Expert Rev. Clin. Immunol. 6, 29–39 20383888 10.1586/eci.09.60

[56] Stephens R. W. Pöllänen J. Tapiovaara H. Woodrow G. Vaheri A. (1991) Stimulation of cell surface plasminogen activation by heparin and related polyionic substances. Semin. Thromb. Hemost. 17, 201–209 1839080 10.1055/s-2007-1002610

[57] Hall S. W. Humphries J. E. Gonias S. L. (1991) Inhibition of cell surface receptor-bound plasmin by α2-antiplasmin and α2-macroglobulin. J. Biol. Chem. 266, 12329–12336 1712017

[58] Chyung Y. Vince B. Iarrobino R. Sexton D. Kenniston J. Faucette R. TenHoor C. Stolz L. E. Stevens C. Biedenkapp J. Adelman B. (2014) A Phase 1 study to assess the safety, tolerability, pharmacokinetics and pharmacodynamics of DX-2930, a human antibody inhibitor of plasma kallikrein under investigation for long-term prophylaxis of hereditary angioedema. Ann. Allergy Asthma Immunol. 10.1016/j.anai.2014.05.028 24980392

[59] Marlar R. A. Kressin D. C. Madden R. M. (1993) Contribution of plasma proteinase inhibitors to the regulation of activated protein C in plasma. Thromb. Haemost. 69, 16–20 8383358

[60] Nilsson T. Wiman B. (1983) Kinetics of the reaction between human C1-esterase inhibitor and C1r or C1s. Eur. J. Biochem. 129, 663–667 6297891 10.1111/j.1432-1033.1983.tb07100.x

[61] Wuillemin W. A. Eldering E. Citarella F. de Ruig C. P. ten Cate H. Hack C. E. (1996) Modulation of contact system proteases by glycosaminoglycans. Selective enhancement of the inhibition of factor XIa. J. Biol. Chem. 271, 12913–12918 8662679 10.1074/jbc.271.22.12913

[62] Pixley R. A. Schapira M. Colman R. W. (1985) The regulation of human factor XIIa by plasma proteinase inhibitors. J. Biol. Chem. 260, 1723–1729 2578463

[63] Brown E. W. Ravindran S. Patston P. A. (2002) The reaction between plasmin and C1-inhibitor results in plasmin inhibition by the serpin mechanism. Blood Coagul. Fibrinolysis 13, 711–714 12441910 10.1097/00001721-200212000-00007

[64] Cugno M. Bos I. Lubbers Y. Hack C. E. Agostoni A. (2001) *In vitro* interaction of C1-inhibitor with thrombin. Blood Coagul. Fibrinolysis 12, 253–260 11460008 10.1097/00001721-200106000-00005

[65] Heeb M. J. España F. (1998) α2-Macroglobulin and C1-inactivator are plasma inhibitors of human glandular kallikrein. Blood Cells Mol. Dis. 24, 412–419 9851894 10.1006/bcmd.1998.0209

[66] Goettig P. Magdolen V. Brandstetter H. (2010) Natural and synthetic inhibitors of kallikrein-related peptidases (KLKs). Biochimie 92, 1546–1567 20615447 10.1016/j.biochi.2010.06.022 PMC3014083

[67] Memari N. Jiang W. Diamandis E. P. Luo L. Y. (2007) Enzymatic properties of human kallikrein-related peptidase 12 (KLK12). Biol. Chem. 388, 427–435 17391064 10.1515/BC.2007.049

[68] Sulikowski T. Patston P. A. (2001) The inhibition of TNK-t-PA by C1-inhibitor. Blood Coagul. Fibrinolysis 12, 75–77 11229830 10.1097/00001721-200101000-00011

[69] Kruithof E. K. Tran-Thang C. Ransijn A. Bachmann F. (1984) Demonstration of a fast-acting inhibitor of plasminogen activators in human plasma. Blood 64, 907–913 6434006

[70] Porebski G. Reshef A. Moldovan D. (2013) The prophylaxis of hereditary angioedema attacks with recombinant human C1 inhibitor: who will take advantage of the individualized treatment approach? Allergy 68, 1207–1209 24074153 10.1111/all.12165

[71] Woodruff R. S. Xu Y. Layzer J. Wu W. Ogletree M. L. Sullenger B. A. (2013) Inhibiting the intrinsic pathway of coagulation with a factor XII-targeting RNA aptamer. J. Thromb. Haemost. 11, 1364–1373 23692437 10.1111/jth.12302 PMC3816843

[72] Woodruff R. S. Sullenger B. Becker R. C. (2011) The many faces of the contact pathway and their role in thrombosis. J. Thromb. Thrombolysis 32, 9–20 21404067 10.1007/s11239-011-0578-5

[73] Bird J. E. Smith P. L. Wang X. Schumacher W. A. Barbera F. Revelli J. P. Seiffert D. (2012) Effects of plasma kallikrein deficiency on haemostasis and thrombosis in mice: murine ortholog of the Fletcher trait. Thromb. Haemost. 107, 1141–1150 22398951 10.1160/th-11-10-0682

[74] Renné T. Gruber A. (2012) Plasma kallikrein: novel functions for an old protease. Thromb. Haemost. 107, 1012–1013 22552331 10.1160/TH12-04-0264

[75] Revenko A. S. Gao D. Crosby J. R. Bhattacharjee G. Zhao C. May C. Gailani D. Monia B. P. MacLeod A. R. (2011) Selective depletion of plasma prekallikrein or coagulation factor XII inhibits thrombosis in mice without increased risk of bleeding. Blood 118, 5302–5311 21821705 10.1182/blood-2011-05-355248 PMC4425441

[76] Crosby J. R. Marzec U. Revenko A. S. Zhao C. Gao D. Matafonov A. Gailani D. MacLeod A. R. Tucker E. I. Gruber A. Hanson S. R. Monia B. P. (2013) Antithrombotic effect of antisense factor XI oligonucleotide treatment in primates. Arterioscler. Thromb. Vasc. Biol. 33, 1670–1678 23559626 10.1161/ATVBAHA.113.301282 PMC3717325

[77] Langhauser F. Göb E. Kraft P. Geis C. Schmitt J. Brede M. Göbel K. Helluy X. Pham M. Bendszus M. Jakob P. Stoll G. Meuth S. G. Nieswandt B. McCrae K. R. Kleinschnitz C. (2012) Kininogen deficiency protects from ischemic neurodegeneration in mice by reducing thrombosis, blood-brain barrier damage, and inflammation. Blood 120, 4082–4092 22936662 10.1182/blood-2012-06-440057 PMC3543983

[78] Girolami A. Scarparo P. Candeo N. Lombardi A. M. (2010) Congenital prekallikrein deficiency. Expert Rev. Hematol. 3, 685–695 21091145 10.1586/ehm.10.69

[79] Chen V. B. Arendall W. B. 3rd Headd J. J. Keedy D. A. Immormino R. M. Kapral G. J. Murray L. W. Richardson J. S. Richardson D. C. (2010) MolProbity: all-atom structure validation for macromolecular crystallography. Acta Crystallogr. D. Biol. Crystallogr. 66, 12–21 20057044 10.1107/S0907444909042073 PMC2803126

